# Using the Theory of Planned Behavior to Understand Factors Influencing South Asian Consumers’ Intention to Seek Pharmacist-Provided Medication Therapy Management Services

**DOI:** 10.3390/pharmacy7030088

**Published:** 2019-07-11

**Authors:** Shaquib Al Hasan, Jagannath Mohan Muzumdar, Rajesh Nayak, Wenchen Kenneth Wu

**Affiliations:** Department of Pharmacy Administration and Public Health, College of Pharmacy and Health Sciences, St. John’s University, 8000 Utopia Parkway, Jamaica, NY 11439, USA

**Keywords:** intention, medication therapy management, pharmacy services, South Asian, theory of planned behavior.

## Abstract

The study purpose was to use the theory of planned behavior to understand factors influencing South Asian consumers’ intention to seek pharmacist-provided medication therapy management services (MTMS). Specific objectives were to assess effects of attitude, subjective norm (SN), perceived behavioral control (PBC), and socio-demographics on South Asian consumers’ intention to seek MTMS. Participants who were ≥18 years of age, of South Asian origin, with a previous visit to a pharmacy in the US for a health-related reason, and with ability to read and comprehend English were recruited from independent pharmacies in New York City. Responses were obtained through a self-administered survey. Descriptive statistics were performed, and multiple linear regression analysis was conducted to assess the study objective. SPSS was used for data analyses. Out of 140 responses, 133 were usable. Mean scores (standard deviation) were 4.04 (0.97) for attitude, 3.77 (0.91) for SN, 3.75 (0.93) for PBC, and 3.96 (0.94) for intention. The model explains 80.8% of variance and is a significant predictor of intention, F (14,118) = 35.488, *p* < 0.05. While attitude (β = 0.723, *p* < 0.05) and PBC (β = 0.148, *p* < 0.05) were significant predictors of intention, SN (β = 0.064, *p* = 0.395) was not. None of the socio-demographics were significant predictors of intention. Strategies to make South Asians seek MTMS should focus on creating positive attitudes and removing barriers in seeking MTMS.

## 1. Introduction

Drug-related problems (DRPs), such as adverse drug reactions, drug interactions, poor adherence etc., significantly affect morbidity and mortality rates and contribute to rising health care expenditures [1,2]. As healthcare professionals, pharmacists can identify, prevent, and resolve DRPs by helping patients understand and self-manage their medications [3,4]. Emphasizing these professional roles of pharmacists, the Centers for Medicare and Medicaid Services (CMS) recognized medication therapy management services (MTMS) under the Medicare Modernization Act of 2003 [5,6]. Medication therapy management (MTM) has been defined as a “distinct service or group of services that optimize therapeutic outcomes for individual patients” [7]. MTM services (MTMS) typically include collecting medical and drug histories from patients, patient education, comprehensive medication review, medication monitoring, and provider outreach to convey recommendations for adjustments to drug therapy when necessary [8,9,10]. Pharmacist-provided MTMS helped improve clinical outcomes such as blood pressure, HbA1c, LDL cholesterol [11]; humanistic outcomes such as medication adherence, patient knowledge, patient satisfaction [11,12]; and economic outcomes such as positive return on investment from MTMS [12].

Previous studies on MTMS mostly focused on healthcare professionals [13,14] and patients [13,15,16,17,18,19,20,21,22,23,24,25,26]. In the literature reviewed, patient-focused studies regarding pharmacist-provided MTMS explored patients’ awareness about MTM [15], attitude toward MTMS [16], perceptions and expectations about MTMS [17], perception about pharmacists’ role [15], perceived medication-related needs [22], patient perceived benefits/values [18], interest or willingness to receive MTMS [20], willingness to pay for MTMS [18], satisfaction with MTMS [21], barriers in receiving MTMS [22], effective strategies for marketing MTM [22], and impact of promotional strategies on patient acceptance of MTMS [24].

However, these patient-focused studies also had their own set of limitations, such as limited sample size [15,21,24], lack of generalizability of study findings [15,17,18,22], low response rate [17,24], and selection bias [21]. In addition, two major limitations are demographics of study participants and lack of theoretical approach in study design. Respondents in previous patient-focused studies were predominantly white [15,18,20]. Their responses might not truly represent opinions of the non-white population, particularly the South Asian population. The South Asian population (people from India, Pakistan, Bangladesh, Nepal, Sri Lanka, Bhutan, and the Maldives) is one of the fastest growing immigrant communities in the United States (US). Members of this racial group have their own social and cultural characteristics, such as language, religion, family ties etc., that may influence their health behavior and healthcare decision making [27,28]. It has been reported that South Asians in the US face various barriers including language, communication, and cultural barriers in accessing health care services [28]. Effective delivery of culturally and linguistically appropriate services can help pharmacists provide better services to this group. In order to design and deliver pharmacy services such as MTMS in a culturally competent way, it is important to understand what influences the intention of South Asians in the US to seek specialized pharmacy services like MTMS. The second major limitation in past literature was the absence of a theoretical framework to explain factors influencing the intention of patients to seek or receive MTMS. Understanding behavior change theories and using them skillfully in research and practice can help design better interventions for patients [29]. This study used the theory of planned behavior (TPB) as a theoretical framework which has been used extensively to explain and predict behavioral intentions and health behaviors including health services utilization [30]. The purpose of this study was to use the theory of planned behavior (TPB) to understand factors influencing intention of South Asian consumers to seek pharmacist-provided medication therapy management (MTM) services. Based on the TPB model, specific study objectives were to assess the influence of South Asian consumers’ (1) attitude on their intention to seek pharmacist-provided MTM services, (2) subjective norm on their intention to seek pharmacist-provided MTM services, (3) perceived behavioral control on their intention to seek pharmacist-provided MTM services, and (4) external variables such as age, gender, income, education, number of medications etc. on their intention to seek pharmacist-provided MTM services.

## 2. Background Literature and Theoretical Framework

The theory of planned behavior is an individual level health behavior theory which hypothesizes that intention is a precursor of actual behavior. According to TPB, intention is influenced by attitude towards performing a behavior, subjective norm associated with the behavior, and perceived control over the behavior [30].

Attitude has been defined as an individual’s positive or negative feelings about performing a behavior [31]. Past application of TPB found that attitude was a significant predictor of pharmacists’ intent to provide MTMS [31], community pharmacists’ intention to utilize a prescription drug monitoring program [32], pharmacists’ intention to report serious adverse drug events (ADEs) to the Food and Drug Administration (FDA) [33], and medical students’ intention to improve oral hygiene [34]. Based on the research cited above, it was hypothesized that

**Hypothesis** **1** **(H1).** *South Asian consumers’ attitude toward seeking MTM services would be a significant predictor of their intention to seek MTM services.*

Subjective norm is an individual’s perception of whether people important to the individual think the behavior should be performed [31]. Past TPB research found that subjective norm was a significant predictor of intention in both pharmacists and consumers. Previous research on pharmacists found that subjective norm was a significant predictor of pharmacists’ intention to provide MTMS [31], intention to utilize a prescription drug monitoring program [32], and intention to report serious ADEs to the FDA [33]. Previous research on consumers found that subjective norm was a significant predictor of consumers’ intention to adopt Pharmacy Value Added Services (PVAS) [35]. Based on the research cited above, the corresponding hypothesis was

**Hypothesis** **2** **(H2).** *South Asian consumers’ subjective norm associated with seeking MTM services would be a significant predictor of their intention to seek MTM services.*

Perceived behavioral control reflects a person’s beliefs as to how easy or difficult it will be to perform the behavior [36]. Like subjective norm, perceived behavioral control was also found to be a significant predictor of intention in both pharmacists and consumers. Perceived behavioral control was found to be a significant predictor of pharmacists’ intent to provide MTMS [31], pharmacists’ intention to utilize a prescription drug monitoring program [32], as well as consumers’ intention to adopt Pharmacy Value Added Services (PVAS) [35]. In the study by Tan et al., perceived behavioral control was found to be the most influential predictor among all TPB constructs in building intent of consumers to adopt PVAS [35]. Based on the research cited above, it was hypothesized that

**Hypothesis** **3** **(H3).** *South Asian consumers’ perceived behavioral control in seeking MTM services would be a significant predictor of their intention to seek MTM services.*

As per TPB, other external variables such as age, gender, type of disease etc., can influence attitude, subjective norm, and perceived behavioral control, and in turn can influence intention to perform certain behavior [30]. In a previous study, age and type of health problems were correlated with willingness to pay for MTMS [18]; gender, income, education, number of health problems, and previous work experience in the healthcare profession were associated with willingness to accept pharmacist-provided MTMS [20]. Among individuals from different cultural groups, length of time in the United States and socio-economic status influenced their beliefs about health, disease, and treatment [37]. Previous research found that gender, education level, and race were significant predictors of the use of family, friends, and co-workers as a source of health information by US adults [38]. Based on this literature, it was hypothesized that

**Hypothesis** **4** **(H4).** *South Asian consumers’ socio-demographic characteristics and other external variables would be significant predictors of their intention to seek MTM services.*

**Figure 1:**
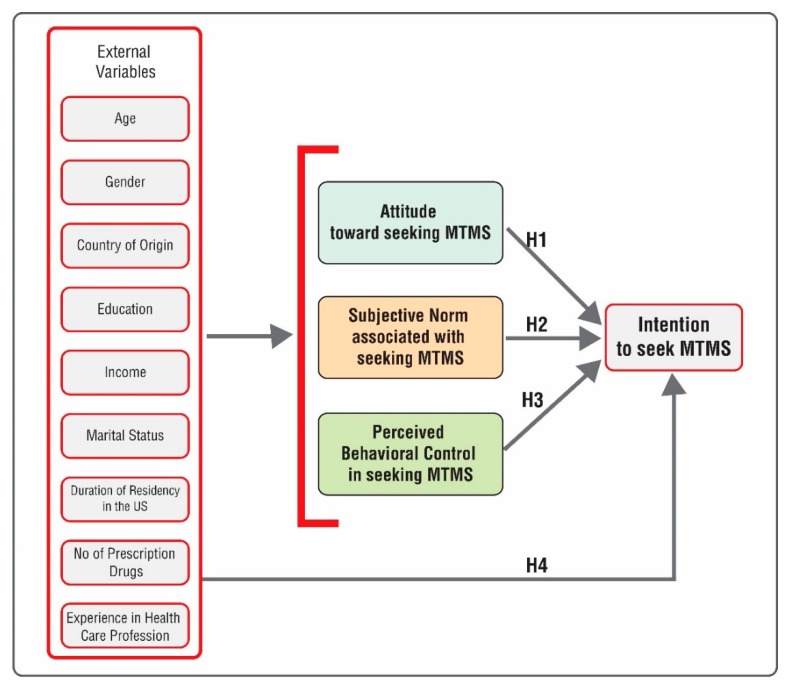
Proposed model of the theory of planned behavior in predicting intention to seek medication therapy management services (MTMS). *Source: Modified from K. Glanz, B. K. Rimer, and K. Viswanath, Health Behavior and Health Education: Theory, Research, and Practice. John Wiley & Sons, pp. 70, 2008.*

A summary of these hypotheses has been presented in Figure 1.

## 3. Methods

### 3.1. Study Design

A cross-sectional, non-experimental, quantitative research design with a self-administered survey approach was used in order to achieve the study objectives.

### 3.2. Target Population and Participant Recruitment

Study participants for survey research were recruited from neighborhoods in New York City. New York City is one of the most diverse cities in the US with a population of different racial backgrounds including South Asians [39]. Moreover, New York State has the second largest concentration of South Asians in the US following California [28]. About 72% of South Asians in New York State resides in New York City [28]. Participants were recruited from New York City neighborhoods of Jamaica Hills and Jackson Heights in Queens, and Ozone Park in Brooklyn where the South Asian population was the most prevalent during the study period. A non-probability convenient sampling was followed for convenience and ease of access to participants. Upon approval from the human subjects review committee, pharmacists in independent pharmacies located in Jackson Heights-Elmhurst, Jamaica Hills, and Ozone Park in New York City were contacted by the primary researcher to inform them about the study purpose and study design, and to seek permission to use their pharmacy premises as the study setting for data collection. Three out of seven pharmacies which were contacted allowed the researcher to use their premises as the study setting and for conducting surveys among consumers visiting their pharmacies. Permissions from the interested pharmacies were taken through signed consent letters.

Study participants were South Asian consumers visiting these three pharmacies. Participants were included in the study if they were of South Asian origin, were at least 18 years of age, had previously visited a pharmacy in the United States for a health related issue, and were able to read and comprehend English. The target sample size calculated for this study was 154 participants. This target sample size was determined from past literature describing methods for sample size calculations required for data analysis techniques (multiple linear regression and factor analysis) used in this study [40,41,42].

### 3.3. Survey Development and Administration

A 28-item survey was developed for data collection. Survey items were developed to measure attitude (five items), subjective norm (four items), perceived behavioral control (five items), and intention (five items). These items were scored on a 5-point Likert scale with possible answers being strongly disagree, disagree, neutral, agree, and strongly agree. Items related to socio-demographic characteristics—age, gender, country of origin, highest level of education, annual household income, marital status, duration of residency in the US, number of medications being taken, and work experience as a health care professional (9 items)—were also included. No personal identifying information of participants was collected to ensure anonymity of participants.

Items assessing attitude and intention were based on the core elements of MTMS (medication therapy review, medication-related action plan, intervention and/or referral, and follow-up) as jointly reported by the American Pharmacists Association (APhA) and the National Association of Chain Drug Stores (NACDS) Foundation [7]. Items from a previous TPB study on pharmacist intention to provide MTMS were modified to develop items assessing subjective norm in this study [31]. Items to assess the perceived behavioral control were developed using past literature on TPB questionnaire construction by Ajzen [43]. Patient-reported barriers to receive MTMS, such as inability to fix appointments due to patients’ busy schedule; inaccessibility to pharmacist (location, parking) and time of day/week reported in past literature were considered while developing items to assess perceived behavioral control [13,22].

Face validity of the survey instrument was done with the help of graduate students and three faculty members having expertise in research methods, survey methodology, and medication therapy management services (MTMS). The survey was approved by the institutional review board.

Upon approval from the human subjects review committee, the survey was pilot tested on a convenience sample of consumers visiting the selected pharmacies between October 2018 and November 2018. The primary researcher approached consumers visiting respective pharmacies and requested for their voluntary participation in the study. The study purpose, procedure of the study, and voluntary nature of participation were explained to the consumers. Interested participants provided their voluntary consent through a participant invitation letter and informed consent form. Consumers who agreed to participate in the study were provided the study survey. A total of 30 surveys were completed in the pilot study as recommended by Viechtbauer et al. [44]. It took approximately 6–10 min for each participant to complete the survey.

Necessary changes were made to the original survey based on the findings from the pilot study. After the pilot study, two items on the intention scale were merged into one item and one item from demographic information was deleted to keep the survey concise. Items asking for previous work experience in the health care profession were moved towards the end of the survey so that participants who did not work in the health care profession did not feel offended in the middle of completing the survey. Moreover, wording for some of the items was changed and examples were provided for some items in the survey to make sure that participants understood the survey items easily. The revised survey was submitted to the human subjects review committee as an amendment to the original Institutional Review Board (IRB) application.

Upon getting the approval for the final survey from the human subjects review committee, data was collected for the main study from mid November 2018 to the end of February 2019. It took approximately 4–8 min for each participant to respond to the survey questionnaire in the main study. The response time for each participant for the main study (4–8 min) was less compared to the pilot study (6–10 min) as the survey instrument for the main study was more easily understandable to participants due to the reduction of the number of items, changes in wording, and addition of examples in the revised survey instrument. To reduce the selection bias, every alternative consumer was requested for participation in the study. A total of 140 consumers participated in this study.

### 3.4. Data Analysis

Prior to data analysis, completeness of survey responses was checked. Any missing data for study variables of a respondent was replaced with an average score of other available items’ responses of the scale within that respondent [45]. Construct validity for survey items was confirmed through factor analysis using principal component analysis with varimax rotation. Items with factor loadings less than 0.60 were considered for deletion. Following the factor analysis, the internal consistency of measures was tested using Cronbach’s alpha. As suggested in the literature, a Cronbach’s alpha of 0.70 or higher was considered as an acceptable reliability coefficient [46].

Socio-demographic characteristics of the study sample were analyzed using descriptive statistical methods. Multiple linear regression analysis was used to assess the influence of independent variables on the dependent variable [47]. In multiple linear regression, attitude, subjective norm, perceived behavioral, and socio-demographic variables were independent variables. Intention was the dependent variable. Before running multiple regression, all of the socio-demographic variables were recoded as dichotomous variables. If the *p*-value was <0.05, the independent variable(s) was considered significant predictor(s) of the intention to seek MTMS. All statistical analyses were performed using IBM SPSS Statistics Version 23.0 (IBM Corp., New York, NY, USA, 2015).

## 4. Results

Out of 140 surveys received, 133 surveys were usable for the study. Seven surveys were not used as six of them had incomplete response to items related to TPB constructs and one did not have demographic information.

### 4.1. Socio-Demographic Characteristics

The majority of the participants were male (71.4%), were 18–45 years old (72.9%), were married or in a domestic relationship (62.4%), had a bachelor’s degree or higher (51.1%), had an annual income less than $35,000 (54.9%), were living in the US for at least 5 years (63.9%), and had no experience in the health care profession (88.0%), as shown in Table 1.

### 4.2. Validity and Reliability of Survey Items

Based on the findings from factor analysis, two items from the initial five items assessing attitude and one item from the initial four items assessing subjective norm were deleted. One of the items for the subjective norm scale was retained in spite of having a factor loading less than 0.60 (0.48) as it is recommended to keep at least three items for each component [48]. No item was deleted from the set of items assessing perceived behavioral control and intention. Finally, data analysis was done on three items for the attitude scale, three items for the subjective norm scale, five items for the perceived behavioral control scale, and five items for the intention scale, as shown in Table 2.

Cronbach’s alpha for the entire survey (excluding sociodemographic variables) was 0.96. Cronbach’s alpha values for attitude, subjective norm, perceived behavioral control, and intention were found to be 0.88, 0.88, 0.93, and 0.93, respectively, as shown in Table 2. Cronbach’s alpha values greater than 0.70 for all of the constructs indicate attitude, subjective norm, perceived behavioral control, and intention scales to be reliable multi-item measures.

### 4.3. Descriptive Statistics for TPB Constructs

Descriptive statistics for TPB constructs have been presented in Table 2. Participants had a mean attitude score of 4.04 with a standard deviation of 0.97. A mean score of 3.77 was found for subjective norm with a standard deviation of 0.91. The lowest mean score of 3.75 with a standard deviation of 0.93 was found for perceived behavioral control based on three items. Finally, a mean score of 3.96 with a standard deviation of 0.94 was found for intention. These mean scores were computed on a Likert scale of 1 to 5 with 1 representing “strongly disagree” and 5 representing “strongly agree”.

### 4.4. Item-Wise Descriptive Statistics for TPB Constructs

Descriptive statistics for individual items including the percentage of respondents having a positive response (either agree or strongly agree), neutral response, and negative response (disagree or strongly disagree) for each statement have been presented in Table 3.

In terms of attitudes toward seeking MTMS, consumers mostly had positive responses to the items measuring attitude towards seeking MTMS. More than 80% of respondents agreed that pharmacists’ advice on how to avoid medication-related problems would be helpful for them, pharmacists’ guidance on self-management of medications would be helpful for them, and pharmacists’ follow-up to ensure patients’ adherence to medications would be good for them. Participants had the most positive attitude towards pharmacists’ guidance on self-management of medications. A total of 88% of the respondents agreed that pharmacists’ guidance on self-management of medications would be helpful for them. On the other hand, respondents had the maximum disagreement with pharmacists’ guidance on avoiding medication-related problems. A total of 14% of the respondents disagreed to the statement that pharmacists’ advice on how to avoid medication-related problems would be useful for them. A total of 84% of the respondents agreed to the statement that pharmacists’ follow-up to ensure patients’ adherence to medications would be good for them.

For subjective norm, more consumers (80%) agreed that family members would support them in taking MTM services than agreed that friends and people important in community would support them in taking MTM services. However, 68% of respondents still agreed that friends would encourage them and 69% of respondents agreed that people important in community would support them in seeking MTM services.

In terms of perceived behavioral control, 80% of respondents believed that they were confident that they would be able to take MTM services. At least 70% of respondents believed that they could easily meet their pharmacist to take his/her services, they should be able to take MTM services without any difficulty, and taking pharmacy services was entirely within their control. However, this percentage was lower in the case of having enough time for taking MTM services. More than one third (37%) of the respondents did not feel (18% disagreed and 19% were neutral) that they had enough time for taking pharmacist-provided MTM services.

Most of the respondents had positive intention toward seeking MTM services. More than 80% of respondents had positive intention to take pharmacists’ follow-up service to ensure adherence to medications. More than 80% of respondents had positive intention to take services in which their pharmacists would review medicines and advise on how to avoid medication-related problems, would educate them on self-management of medications, and educate them on lifestyle changes to help them self-manage their diseases. On the contrary, respondents had the least positive intention to seek service of pharmacists’ recommendations to physicians about medications. A total of 22% of respondents did not feel (14% disagreed and 8% were neutral) that they had intention to seek services where pharmacists would recommend physicians for better medications. Nevertheless, 77% of respondents had positive intention to seek services where pharmacists would recommend physicians for better medications.

### 4.5. Multiple Linear Regression Predicting Intention

Hypothesis testing was done by multiple linear regression. Hierarchical multiple regression was used for the study variables to find out the relative contribution of socio-demographic variables versus other TPB constructs (attitude, subjective norm, and perceived behavioral control) in predicting intention. In step 1, attitude, subjective norm, and perceived behavioral control were included as predictor variables (Model 1). In step 2, along with TPB constructs (attitude, subjective norm, and perceived behavioral control), socio-demographic variables were entered as predictors of intention (Model 2). Table 4 presents model summary for Model 1 and Model 2.

Model 1 had an R^2^ value equal to 0.791 and Model 2 had an R^2^ value equal to 0.808. It means that 79.1% of the variance in intention was explained by the independent variables in Model 1 and 80.8% of the variance in intention was explained by the independent variables in Model 2. Entry of socio-demographic variables (Model 2) resulted in a change in R^2^ of 0.017. This means that entry of socio-demographic variables increased the explained variance in intention by only 1.7% to a total of 80.8%. This increase was not statistically significant by the F change test, F (11,118) = 0.959, *p* = 0.487. These findings suggest that the first set of predictor variables other than socio-demographic variables (attitude, subjective norm, and perceived behavioral control) were a more powerful set of predictors than socio-demographic variables and inclusion of socio-demographic variables did not increase explanatory power significantly.

#### 4.5.1. Influence of Attitude on Intention

The first specific objective of the study was to assess the influence of South Asian consumers’ attitude toward seeking MTMS on their intention to seek MTMS. The regression analysis for Model 1 found that attitude was a statistically significant predictor of intention, β = 0.732, t = 11.258, *p* < 0.05, as shown in Table 5. The regression analysis for Model 2 also found that attitude was a statistically significant predictor of intention, β = 0.723, t = 10.303, *p* < 0.05, as shown in Table 5. This means attitude was a significant predictor of intention (*p* < 0.05) for both models.

#### 4.5.2. Influence of Subjective Norm on Intention

The second study objective was to assess the influence of South Asian consumers’ subjective norm associated with seeking MTM services on their intention to seek MTMS. The regression analysis for Model 1 found that subjective norm was not a statistically significant predictor of intention, β = 0.042, t = 0.587, *p* = 0.558, as shown in Table 5. The regression analysis for Model 2 also found that subjective norm was not a statistically significant predictor of intention, β = 0.064, t = 0.854, *p* = 0.395, as shown in Table 5. This means subjective norm was not a significant predictor of intention (*p* < 0.05) for both models.

#### 4.5.3. Influence of Perceived Behavioral Control on Intention

The third study objective was to assess the influence of South Asian consumers’ perceived behavioral control in seeking MTM services on their intention to seek MTMS. The regression analysis for Model 1 found that perceived behavioral control was a statistically significant predictor of intention, β = 0.169, t = 2.738, *p* < 0.05, as shown in Table 5. The regression analysis for Model 2 found that perceived behavioral control was a statistically significant predictor of intention, β = 0.148, t = 2.224, *p* < 0.05, as shown in Table 5. This means perceived behavioral control was a significant predictor of intention (*p* < 0.05) for both models.

#### 4.5.4. Influence of Socio-Demographic Characteristics on Intention

The fourth specific study objective was to assess the influence of South Asian consumers’ socio-demographic characteristics on their intention to seek MTMS. Prediction by none of these socio-demographic variables was found to be significant at a level of significance of 0.05, as shown in Table 5.

## 5. Discussion

To the researcher’s knowledge, this is the first consumer/patient focused study regarding MTMS that used TPB. However, the results found in this study may be influenced by the demographics of study participants. The study group was quite skewed toward younger people, with only 6.8% of participants having an age of 65 years or more, whereas 16% of the total US population are 65 years or older [49]. A total of 51 out of 133 respondents (38.3%) reported no prescription medications taken daily and these respondents may not perceive a need for MTM at all. While 53% of the total South Asian population in the US are male [28], 71.4% of study participants in this study were male. This gender skewing (95 males to 38 females) could also have influenced responses. The educational data showed 51.1% with bachelor or higher degrees while this percentage for South Asians in the entire US is 59% [28]. These data indicate that findings from this study may not be generalizable to the entire US population. Due to skewness in demographics of study participants, results may slightly vary from opinions of the total South Asian population in the US.

The purpose of this study was to use the theory of planned behavior (TPB) to understand factors influencing intention of South Asian consumers to seek pharmacist-provided medication therapy management (MTM) services. The study results found that the overall attitude, subjective norm, perceived behavioral control, and intention of South Asian consumers to seek MTMS was positive. This is consistent with findings from previous studies regarding MTMS where patients had a positive attitude towards receiving MTMS, were willing to take MTMS, and recognized pharmacists as potential providers of MTMS [18]. The majority of respondents had positive intention to seek each component of MTMS, as shown in Table 3. However, respondents had the least agreement with the statement that they would be willing to take MTM services where pharmacists will recommend physicians for safer or more effective medication. This finding is consistent with findings from the study by Brown et al. [20] where consumers were not favoring services that involve contacting their physicians for recommending safer or more effective medications.

The study findings indicate that South Asian consumers’ intention to seek MTMS was driven by attitude and perceived behavioral control. This finding is consistent with previous research regarding applications of TPB in health-related behaviors [32,33,34,50,51]. However, considering the influence of subjective norm, this finding is slightly different from TPB research assessing intention to receive pharmacy services [35]. Details on the influence of attitude, subjective norm, perceived behavioral control, and socio-demographic characteristics on intention have been described below:

### 5.1. Influence of Attitude on Intention

South Asian consumers’ attitude towards seeking MTM services was found to be a significant and strongest predictor of their intention to seek MTM services. This finding is consistent with previous TPB research assessing pharmacists’ intent to provide MTMS [31], community pharmacists’ intention to utilize a prescription drug monitoring program [32], pharmacists’ intention to report serious ADEs to the FDA [33], patients’ intention to participate in physical exercise [52], and medical students’ intention to improve oral hygiene [34].

More than 80% of respondents had a positive attitude toward seeking each component of MTMS, as shown in Table 3. This finding is consistent with previous findings from the study by Schultz et al., aimed at determining patient-perceived value of MTMS. Schultz et al. found that MTMS was considered to be valuable, satisfactory, and financially beneficial to patients [19]. In this current study, respondents were the most positive with services involving the pharmacist’s educating self-management of medications and were the least positive with services involving the pharmacist’s guidance on managing drug-related problems (DRPs). Similar results were also evident in past research [16]. Like this current study, in a study by Doucette et al., services involving explaining how to use medications had the highest mean score [23].

To strengthen South Asian consumers’ intention to seek MTM services, pharmacists should focus on creating and maintaining positive attitude toward MTM services. Previous research found that participants had a positive attitude toward pharmacists in terms of pharmacists’ knowledge and problem solving capability [22] and pharmacists were believed to be good candidates to provide MTM services [15]. Patients receiving MTM services also identified their MTM pharmacist as a supporter, advocate, confidant, resource for education, and coordinator of medications [19]. However, perception of pharmacists revolved around the medication dispensing function [15] and patients were skeptical about pharmacists’ interaction with patients [22]. This perception may have a negative influence on attitudes towards seeking pharmacist-provided services. It is a matter of further investigation of which factors influence South Asian consumers’ attitude toward seeking pharmacist-provided services. To create a positive attitude toward seeking pharmacist-provided MTMS, pharmacists have to create a perception of providing personalized information about medications and advising South Asian patients on medications through interaction with South Asian patients beyond traditional dispensing. During this current study, it has been found that pharmacy technicians have the opportunity to interact with patients. Along with pharmacists, pharmacy technicians may also contribute to creating a positive attitude towards pharmacy and pharmacy services though positive interactions with South Asian patients. A previous study found that the perceived importance of pharmacist-provided comprehensive medication reviews was more in patients experiencing comprehensive medication reviews than in patients not experiencing comprehensive medication reviews from pharmacists [23]. In order to increase perceived importance of MTMS, pharmacists can provide MTMS for free or a reduced/discounted price for a short time (like a trial version) to have South Asian patients experience the flavor of MTMS and then for an extended period (like a full version) once South Asian patients have experienced MTMS.

### 5.2. Influence of Subjective Norm on Intention

Subjective norm was not a statistically significant predictor of intention. This finding differs with findings from previous applications of TPB where subjective norm was a significant predictor of pharmacists’ intent to provide MTMS [31], intention to utilize a prescription drug monitoring program [32], and intention to report serious ADEs to the FDA [33]. This may due to the fact that pharmacists felt the competitiveness to provide MTMS seeing other pharmacists providing MTMS. On the other hand, consumers might intend to seek MTMS when they experience certain health conditions or medications.

However, this finding also differs with the result from a consumer-focused study done by Tan et al. using TPB. Malaysian consumers’ subjective norm was found to be a significant predictor of intention to adopt Pharmacy Value Added Services (PVAS) introduced by Malaysia’s Ministry of Health [35]. As described by Fishbein, the degree of influence of attitude, subjective norm, and perceived behavioral control on intention may vary depending on behavior and the population being considered [53]. The difference in population (South Asians in the US vs. Malaysians in Malaysia) and behavior (seeking MTMS vs. adopting PVAS) may explain different degrees of influence of subjective norm on intention.

Considering South Asian consumers typically get their health care related information primarily from family members and physicians, this finding was particularly interesting [27]. This finding also conflicts with previous research where influence of family members was found an important factor for South Asian patients’ health behavior, such as medication adherence among South Asian patients with cardiac diseases [54]. Further studies would be needed to explore why subjective norm did not significantly predict intention of South Asian consumers to seek MTMS.

### 5.3. Influence of Perceived Behavioral Control on Intention

In this study, perceived behavioral control was found to be a statistically significant predictor of intention. This finding is consistent with the results from previous research on pharmacists and consumers where perceived behavioral control was found to be a significant predictor of pharmacists’ intent to provide MTMS [31], pharmacists’ intention to utilize a prescription drug monitoring program [32], as well as consumers’ intention to adopt Pharmacy Value Added Services (PVAS) [35].

In order to increase South Asian consumers’ intention to seek MTMS, focus should be given to minimizing their perceived barriers in seeking MTMS. In this study’s findings, most respondents were confident that they can take pharmacist-provided services. However, a total of 37% of respondents did not feel (18% disagreed and 19% were neutral) that they had enough time for taking pharmacist-provided services, as shown in Table 3. This finding is also consistent with findings from previous studies on patient-reported barriers to receive MTMS [19,22]. This indicates that matching the patient’s convenient time with the MTM pharmacist’s available time would be an important consideration in facilitating the patient’s taking of MTM services. Although inability to fix times with MTM pharmacists due to patients’ busy schedules is a common problem found in previous studies; Moczygemba et al. found that patients were satisfied with pharmacist-provided telephone-based MTM care [21]. In telephone-based MTM care, patients viewed pharmacists as easily accessible and responsive to patients’ problems [21]. In order to match the timing between pharmacists and South Asian patients, pharmacists can consider providing telephone-based MTM care as an alternative option for South Asian patients.

In the previous literature, patient-reported barriers to receive MTMS were patients’ lack of knowledge of MTMS; lack of perceived need for MTMS; out-of-pocket cost; lack of availability of the MTM pharmacist at patients’ convenient time; inability to fix appointments due to patients’ busy schedule; inaccessibility to pharmacist MTM practice due to the location, parking, time of day/week; and fear of obtaining recommendations conflicting with their physician’s plan of care [13,19,22]. Pharmacists can check if these barriers are also South Asian consumers’ barriers to receive MTMS. Pharmacists should keep any such barriers as low as possible so that patients are willing to seek MTMS. Pharmacists should also observe and resolve any cultural barriers (such as language barriers) specifically faced by South Asian consumers in order to strengthen South Asian patients’ intention to seek MTMS.

### 5.4. Influence of Socio-Demographic Characteristics on Intention

In this study, none of the socio-demographic characteristics was found to be statistically significant predictors of intention.

Age was expected to be an important factor influencing intention to seek MTMS. Since elderly people are more prone to have chronic diseases and use multiple medications, they are supposed to need help in managing their medications. Moreover, previous research found a correlation of age with willingness to pay for MTMS [18]. Unexpectedly, age was not a significant predictor of intention to seek MTMS in the South Asian group.

In previous studies, the willingness to accept pharmacist-provided MTMS differed in terms of gender [20]. In the current study, intention to seek MTMS differed in terms of gender. Although the survey was anonymous, a number of females, because of their conservative cultural background, did not respond to the survey during the study. A common response from female consumers for their non-participation in the survey was “I cannot fill up any survey without my husband’s permission”. This non-response has resulted in a decreased proportion of female participants compared to male participants. However, gender was not found to be a significant predictor of intention to seek MTMS.

In previous studies, the willingness to accept pharmacist-provided MTMS differed in terms of education level and income [20]. In the current study, intention to seek MTMS also differed in terms of education and income. Nonetheless, none of the variables were found to be significant predictors of intention to seek MTMS in this particular group. Although married consumers had a mean intention score less than unmarried ones, marital status was not found to be a significant predictor of intention to seek MTMS. 

Duration of residency in the US was included as a socio-demographic predictor variable as consumers may get acquainted with the US health care system and be more aware of health services with the increasing duration of residency in the US. Country of origin was included as socio-demographic variable as culture in different countries can influence health behavior. Neither duration of residency in the US nor country of origin was found to be a significant predictor of intention to seek MTMS.

In past research, consumers’ work experience in the healthcare profession was found associated with their willingness to accept MTM services [20]. It was expected that South Asian consumers’ work experience in the healthcare profession would influence their intention to seek MTMS. Unexpectedly, experience in the healthcare profession had no influence on South Asian consumers’ intention to seek MTMS.

The noteworthy unexpected finding was that number of medications had no statistically significant impact on intention to seek MTMS. It was expected that the more medications one takes, the more would be the need for managing medications and thus, intention to seek MTMS. It is possible that patients are taking medications on an on-demand basis and not on a regular basis, or they are already familiar with medications. That is why they probably did not feel the need for MTMS and had no intention to seek such services. More than one-third (38.3%) of the respondents were taking no prescription drugs. It may be due to the fact that many non-patient consumers visited pharmacies to get medications for their family members. 

## 6. Limitations and Future Research

Like any other research, this study has some limitations, too. Firstly, most of the respondents (76.7%) were of Bangladeshi origin. Demographically, the selected sample as a whole may not represent South Asian consumers in the US. Although the South Asian community is diverse in languages and religious practices, there are many shared social and cultural characteristics among South Asians [28]. Thus, although most of the respondents were of Bangladeshi origin, they still represent South Asians holding those social and cultural characteristics. Secondly, the target sample size could not be reached due to the limited duration of the study period and non-response from South Asian consumers, particularly from females. However, a sample size of 133 used in this study is sufficient for factor analysis for both Model 1 and Model 2 [40,41] and also adequate for multiple linear regression analysis for Model 1 [42]. Only for multiple linear regression analysis in Model 2, a sample size of 133 is less than the recommended sample size of 154 [42]. Thirdly, this study did not consider the actual behavior of consumers, an important construct of TPB, which may vary from intention measured in the study. Fourthly, this study assessed degree of influence of attitude, subjective norm, and perceived behavioral control on intention but did not find out the reasons for such influence. A qualitative study would complement quantitative research in finding out specific reasons for such influence. Finally, although previous research found that disease state, number of health problems, familiarity with medications, and insurance status had correlations with either willing to receive MTMS or willingness to pay for MTMS, these variables were not included in this study. Future research can include these variables.

Future research opportunities include conducting qualitative research to find out the reasons behind the influence of factors such as attitude, subjective norm, and perceived behavioral control on intention to seek pharmacist-provided MTMS. Previous research showed that not all respondents willing to receive MTMS are willing to pay for MTMS [18] and most of the respondents think that insurance companies should cover cognitive pharmacy services [55]. South Asian consumers’ willingness to pay for MTMS can also be studied to assess how payment influences their intention and actual performance of behavior, that is, receiving pharmacist-provided MTM services.

## 7. Conclusions

Overall, South Asian consumers showed a positive attitude, favorable subjective norm, favorable perceived behavioral control, and positive intention to seek MTM services. The TPB was revealed to be a good model to measure and predict South Asian consumers’ intention to seek MTM services. Multiple linear regression analysis showed that attitude and perceived behavioral control were significant predictors of intention (*p* < 0.05), but subjective norm was not a significant predictor of intention. Socio-demographic characteristics did not have a significant effect on intention.

Strategies to make South Asian consumers seek MTM services should focus on enhancing and maintaining positive attitudes by showing expertise in medicines through interactions with South Asian patients, identifying and promoting factors (e.g., pharmacists’ knowledge) responsible for positive attitudes among South Asian patients, promoting perceived facilitators (e.g., easy access) in seeking MTMS, and resolving possible barriers (e.g., timing, language) perceived by South Asian patients in seeking MTMS.

## Figures and Tables

**Table 1:** Socio-demographic characteristics of the study sample (N = 133).

Characteristic	Number	Percent
**Age**		
Between 18 and 30 years	51	38.3
Between 31 and 45 years	46	34.6
Between 46 and 65 years	27	20.3
65 years or more	9	6.8
**Gender**		
Male	95	71.4
Female	38	28.6
**Country of Origin**		
Bangladesh	102	76.7
India Nepal	11 12	8.3 9.0
Pakistan	8	6.0
**Education**		
Less than high school	13	9.8
High school or equivalent	33	24.8
Some college, no degree	10	7.5
Associate degree	9	6.8
Bachelor’s degree	29	21.8
Graduate degree	39	29.3
**Household income**		
Less than $20,000	42	31.6
$20,000 to $34,999	31	23.3
$35,000 to $49,999	23	17.3
$50,000 to $74,999	14	10.5
$75,000 to $99,999	14	10.5
$100,000 or more	9	6.8
**Marital status**		
Single (never married)	46	34.6
Married, or in a domestic partnership	83	62.4
Widowed	2	1.5
Divorced	1	0.8
Separated	1	0.8
**Duration of residency in the US**		
Less than 1 year	2	1.5
1 to 3 years	22	16.5
3 to 5 years	24	18.0
5 to 10 years	43	32.3
10 years or more	42	31.6
**No. of daily prescription drug**		
0	51	38.3
1	30	22.6
2	21	15.8
3	6	4.5
4 or more	25	18.8
**Experience in health care profession**		
Yes	16	12.0
No	117	88.0

**Table 2:** Theory of planned behavior scale constructs used to measure intention to seek MTMS (N = 133).

Construct	No. of Items Retained (No. of Initial Items)	Mean ^a^	Standard Deviation ^a^	Lowest Individual Score	Highest Individual Score	Cronbach’s Alpha
Attitude	3 (5)	4.04	0.97	1.00	5.00	0.88
Subjective norm	3 (4)	3.77	0.91	1.00	5.00	0.88
Perceived behavioral control	5 (5)	3.75	0.93	1.00	5.00	0.93
Intention	5 (5)	3.96	0.94	1.00	5.00	0.93

^a.^ Individual items were measured on a Likert scale (1 = strongly disagree through 5 = strongly agree). Higher scores signify more positive attitudes, more favorable subjective norm, more perceived behavioral control and stronger intention to seek MTMS.

**Table 3:** Survey statements and descriptive statistics by construct (N = 133).

Construct	Statement	Mean (SD) ^a^	Percent Agree ^b^	Percent Neutral	Percent Disagree ^c^
	If pharmacist advises me on how to avoid medicine-related problems such as possible adverse effects or side effects, allergy due to medicine; it will be helpful for me.	3.99 (1.14)	82.0	4.5	13.5
Attitude	If pharmacist guides me on how to handle my medicines by myself (such as taking appropriate action if the dose is missed, using a pill reminder chart for taking medicines timely), it will be helpful for me.	4.11 (1.03)	88.0	3.0	9.0
	If pharmacist communicates with me to ensure that I am taking my medicines according to doctor’s prescription, it will be good for me.	4.02 (1.06)	83.5	4.5	11.3
	If I want to take pharmacy services, my family members will support me.	3.90 (0.92)	79.7	9.0	11.3
Subjective Norm	If I want to take pharmacy services, my friends will support me.	3.75 (1.05)	68.4	20.3	11.3
	People important to me in my community will support me to take pharmacy services.	3.65 (1.05)	69.2	17.3	13.5
	I can easily meet my pharmacist to take his/her services.	3.76 (0.99)	72.9	11.3	15.0
	I am confident that I shall be able to take pharmacy services.	3.92 (0.97)	79.7	10.5	9.8
Perceived Behavioral Control	I shall be able to take pharmacy services without any difficulty.	3.74 (1.06)	72.2	13.5	14.3
	I have enough time for taking services provided by pharmacist.	3.55 (1.16)	63.2	18.8	18.0
	Taking pharmacy services is entirely within my control.	3.77 (1.10)	72.9	12.0	13.5
	I am willing to take the service in which my pharmacist … …will evaluate my medicines and tell me how to avoid medicine-related problems such as possible adverse effects or side effects, allergy due to medicine etc.	3.95 (1.05)	81.2	7.5	11.3
Intention	…will educate me so that I can handle my medicines by myself (such as taking appropriate action if the dose is missed, using pill reminder chart for taking medicines timely).	3.95 (1.08)	83.5	3.8	12.8
	…will educate me on diet, physical activity, smoking, drinking etc. so that I can manage diseases such as diabetes.	3.96 (1.05)	82.0	6.8	11.3
	…will recommend my doctor to change the medicine where there is more effective or safe medicine.	3.86 (1.09)	77.4	8.3	14.3
	…will contact me and give advice to make sure that I am continuing medicines according to my doctor’s prescription.	4.05 (1.04)	86.5	3.8	9.8

SD = standard deviation. ^a^ Coded as 1 = strongly disagree, 2 = disagree, 3 = neutral, 4 = agree, 5 = strongly agree. ^b^ Percent stating they agreed or strongly agreed. ^c^ Percent stating they disagreed or strongly disagreed.

**Table 4:** Model summary of multiple linear regression analysis predicting intention.

Model ^a^	R	R^2^	Adjusted R^2^	Standard Error of Estimate	R^2^ Change	F Change	df1	df2	Sig. F Change
1	0.889 ^b^	0.791	0.786	0.43605	0.791	162.655	3	129	0.000
2	0.899 ^c^	0.808	0.785	0.43680	0.017	0.959	11	118	0.487

^a.^ Dependent variable: intention. ^b.^ Predictors (constant): attitude, subjective norm, perceived behavioral control. ^c.^ Predictors (constant): attitude, subjective norm, perceived behavioral control, age more than 45 years, male gender, married or in a domestic relationship, Bangladeshi origin, Nepali origin, Indian origin, bachelor’s degree or higher, income more than $35,000, duration of residency in the US, daily prescription drug quantity, experience in health care profession.

**Table 5:** Parameter estimates from multiple linear regression analysis predicting intention to seek medication therapy management services (N = 133).

Model	Variable	Unstandardized Coefficients		Standardized Coefficients		
		B	Standard Error	Beta	t Statistic	*P* Value
	Constant	0.274	0.177		1.550	0.124
	Attitude	0.711	0.063	0.732	11.258	0.000 *
1 ^a^	Subjective norm	0.043	0.074	0.042	0.587	0.558
	Perceived behavioral control	0.171	0.063	0.169	2.738	0.007 *
	Constant	0.444	0.239		1.860	0.065
	Attitude	0.703	0.068	0.723	10.303	0.000 *
	Subjective norm	0.067	0.078	0.064	0.854	0.395
	Perceived behavioral control	0.150	0.067	0.148	2.224	0.028 *
	Age	−0.100	0.109	−0.047	−0.918	0.361
	Gender	−0.014	0.099	−0.007	−0.143	0.887
	Bangladeshi Origin	0.042	0.168	0.019	0.251	0.802
2 ^a,b^	Nepali Origin	−0.065	0.209	−0.020	−0.311	0.756
	Indian Origin	0.112	0.214	0.033	0.522	0.602
	Education	−0.031	0.083	−0.016	−0.368	0.714
	Income	0.011	0.092	0.006	0.123	0.902
	Marital Status	−0.138	0.091	−0.071	−1.511	0.133
	Duration of Residency in the US	−0.142	0.092	−0.072	−1.540	0.126
	No of Daily Rx Drug	0.147	0.114	0.066	1.286	0.201
	HCP Experience	0.092	0.123	0.032	0.748	0.456

* Significant predictor of intention at a level of significance of 0.05. ^a.^ Dependent variable: intention. ^b.^ Age was coded as 1 = more than 45 years, 0 = 18 to 45 years; gender was coded as 1 = male, 0 = female; Bangladeshi origin was coded as 1 = Bangladeshi origin, 0 = non- Bangladeshi origin; Nepali origin was coded as 1 = Nepali origin, 0 = non- Nepali origin; Indian origin was coded as 1 = Indian origin, 0 = non-Indian origin; education was coded as 1 = bachelor’s and above, 0 = Less than bachelor’s; income was coded as 1 = $35,000 and above, 0 = up to $34,999; marital status was coded as 1 = married or in a domestic relationship, 0 = other; duration of residency in the US was coded as 1 = more than 5 years, 0 = up to 5 years; daily prescription drug quantity was coded as 1 = 3 or more, 0 = less than 3; health care profession (HCP) experience was coded as 1 = previous experience in health care profession, 0 = no experience in health care profession.
